# Phenylketonuria and type 1 diabetes: a clinical and nutritional challenge in a young adult—a case report

**DOI:** 10.1007/s00592-026-02685-6

**Published:** 2026-06-19

**Authors:** Carmine Piccolo, Sara de Candia, Annalisa Natalicchio, Sergio Di Molfetta, Irene Caruso, Luigi Laviola, Francesco Giorgino, Gian Pio Sorice

**Affiliations:** Department of Precision and Regenerative Medicine and Ionian Area, Section of Internal Medicine, Endocrinology, Andrology and Metabolic Diseases, University of Bari Aldo Moro, Bari, Italy

**Keywords:** Phenylketonuria, Type 1 diabetes, CGM, multidisciplinary approach

## Abstract

**Aims:**

Phenylketonuria and type 1 diabetes are lifelong metabolic disorders requiring complex and potentially conflicting nutritional strategies. Their coexistence is rare, yet management may become particularly challenging during transition from pediatric to adult care. We describe the case of a young adult with phenylketonuria who developed type 1 diabetes.

**Methods:**

A 27-year-old man with longstanding phenylketonuria was referred to an adult metabolic-diabetes center after the diagnosis of type 1 diabetes. Clinical, biochemical, nutritional, and continuous glucose monitoring data were reviewed. The intervention included structured therapeutic education, transition from fixed insulin doses to a dynamic regimen based on carbohydrate counting, and revision of medical nutrition therapy using phenylketonuria-adapted low-protein foods and sugar-free phenylalanine-free amino acid supplements.

**Results:**

At diagnosis, HbA1c was 11.5%, with markedly reduced C-peptide levels and high titer anti-GAD antibodies. Initial diabetes management was associated with poor adherence to the phenylketonuria diet, increased intake of conventional protein sources, and elevated phenylalanine levels. After individualized insulin titration and nutritional intervention, HbA1c improved from 11.5% to 7.8%, phenylalanine levels decreased from 842 to 705 μmol/L, insulin requirement declined from 0.55 to 0.3 IU/kg/day, and continuous glucose monitoring showed improved glycemic control without increased hypoglycemia. The Glycemia Risk Index improved from high-risk Zone E to low-intermediate-risk Zone B.

**Conclusions:**

This case highlights the need for personalized multidisciplinary care integrating continuous glucose monitoring, carbohydrate counting, and phenylketonuria specific nutrition to optimize both metabolic conditions.

## Background

Type 1 diabetes (T1D) and phenylketonuria (PKU) are two distinct metabolic disorders whose co-occurrence presents unique clinical challenges. PKU is a rare inherited metabolic disorder caused by a deficiency in the enzyme phenylalanine hydroxylase, which converts phenylalanine to tyrosine. Lifelong adherence to a strict low-phenylalanine diet is required for patients with PKU to prevent intellectual disability and other serious complications. T1D is an autoimmune condition characterized by the destruction of pancreatic beta cells, leading to absolute insulin deficiency and requiring lifelong insulin therapy. This condition typically develops in childhood or adolescence and affects glucose metabolism, necessitating monitoring of blood sugar levels and carbohydrate intake.

Diabetes and PKU co-occurrence is more common than expected, though comprehensive epidemiological data remains limited. A large German study of 377 adult PKU patients found significantly higher rates of diabetes compared to matched controls, with unspecified diabetes mellitus showing a prevalence ratio of 1.7 and type 2 diabetes mellitus demonstrating a prevalence ratio of 1.3 [1]. This increased prevalence has been replicated in other studies, where diabetes mellitus was identified among the conditions with the highest prevalence ratios (1.6–2.3) in PKU patients compared to controls [1, 2]. The higher prevalence of diabetes in PKU patients has been attributed to the high carbohydrate intake required by the PKU diet, though researchers note that “there is currently no clear evidence that patients with PKU exhibit a higher risk of developing diabetes and most studies only include children or young adults, which may exclude the development timeline of type 2 diabetes mellitus” [1]. As the PKU population ages due to improved treatments, adults with PKU are increasingly at risk for developing age-related conditions including diabetes [2]. In contrast to the documented cases of type 2 diabetes, T1D co-occurring with PKU appears to be much rarer [3], with only case reports in the literature [4, 5].

The clinical management of patients with co-occurring PKU and T1D presents unique challenges that require specialized expertise and careful coordination between multiple therapeutic approaches. The complexity of managing both conditions simultaneously is reflected in research practices, where diabetes is commonly listed as an exclusion criterion in PKU studies, limiting the available evidence base for treating these dual-diagnosis patients. This exclusion practice suggests that researchers recognize the potential confounding effects of diabetes on PKU management and outcomes, but it also negatively impacts on generation of evidence-based guidelines for managing both conditions together.

The primary clinical challenge in managing patients with both PKU and T1D lies in balancing the strict low-phenylalanine diet required for PKU with the glucose management needs of individuals with type 1 diabetes. PKU patients generally consume special low-protein products low in phenylalanine and rich in carbohydrates, which can complicate blood glucose control and the implementation of insulin therapy in those with T1D. Healthcare teams managing these complex cases must include specialists familiar with both conditions, including endocrinologists, metabolic physicians, and specialized dietitians who can handle all sorts of dietary needs. Regular monitoring becomes even more critical, as changes in either condition can affect the management of the other, requiring frequent adjustments of both dietary plans and insulin regimens.

## Case presentation

Herein, we describe the case of a 27-year-old adult with PKU, who had been followed at a pediatric care center and recently developed T1D.

In November 2022, the patient presented to the Emergency Room with occasional hyperglycemia and weight loss. Diabetic ketoacidosis (DKA) was biochemically excluded at onset (based on pH, anion gap, and ketone levels), and no acute precipitating factors, such as infections, were identified. T1D was suspected, and insulin therapy with multiple daily injections was initiated. In December 2022, the patient was admitted to our adult center.

On examination, the patient had a BMI of 19.7 kg/m², normal blood pressure, and a normal ECG. Laboratory results showed glycated hemoglobin (HbA1c) of 11.5%, C-peptide of 0.4 nmol/L, and positive anti-GAD autoantibodies (6.6 UA/mL; normal < 5 UA/mL, Insulin, IA2, and ZnT8 autoantibodies negative), confirming the diagnosis of T1D. Monogenic diabetes was also excluded through genetic testing of HNF4A, GCK, HNF1A, PDX1 AND HNF1B, which revealed no associated mutations. Autoimmunity screening was also performed for the main conditions typically associated with type 1 diabetes, such as thyroiditis and celiac disease, with negative results.

The patient’s PKU management was suboptimal, with serial phenylalanine (Phe) levels (normal range 120–600 µmol/L) and Phe/Tyr ratios consistently above recommended targets. Initially, the patient was advised to follow the PKU diet and was prescribed a continuous glucose monitoring (CGM) system.

**Fig. 1:**
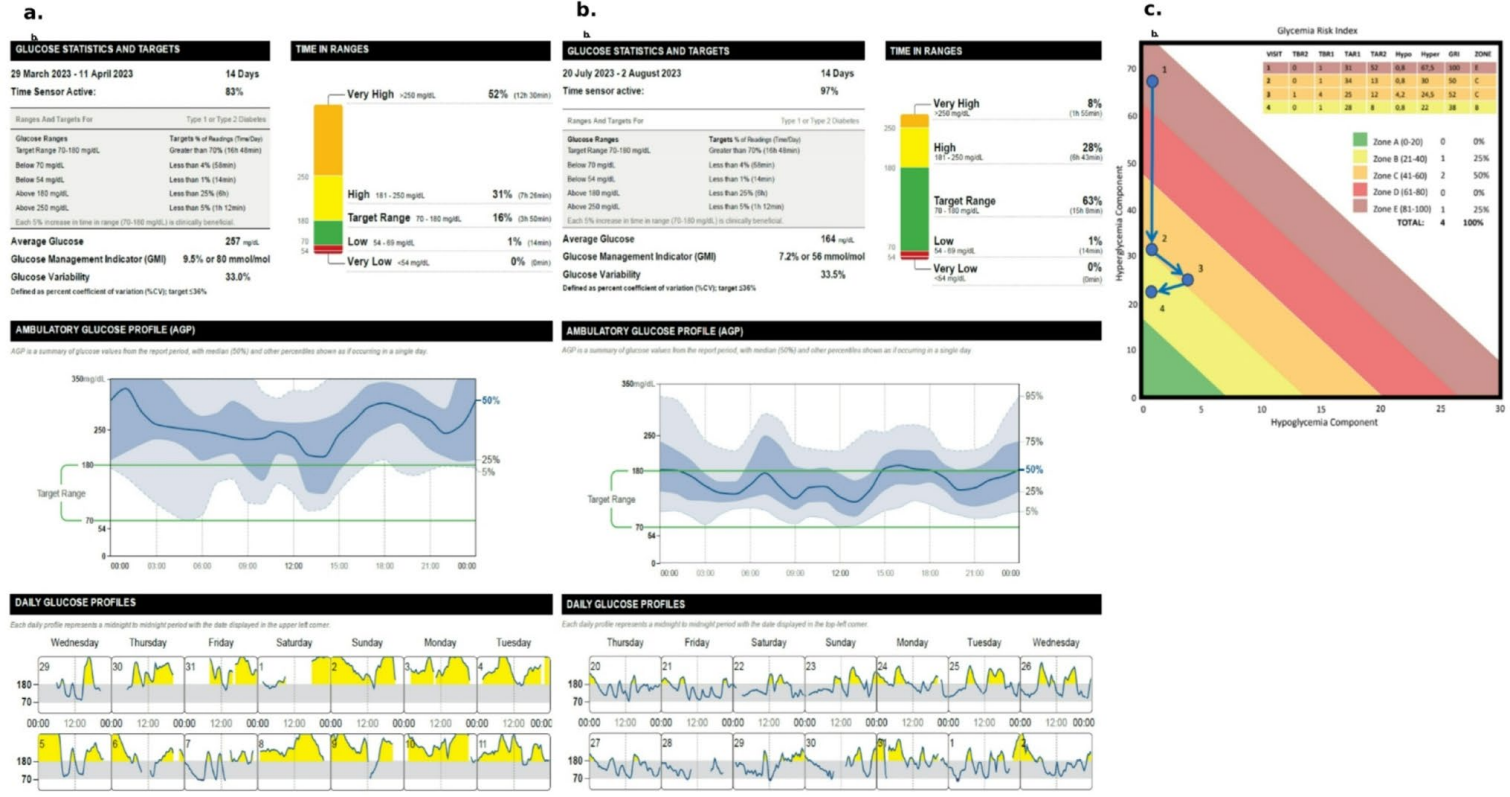
**(a)** Initial Continuous Glucose Monitoring (CGM) ambulatory glucse profile (AGP) showing poor glycemic control. The report from March-April 2023 indicates a Time in Range (70–180 mg/dL) of only 16%, with 31% of TAR level 1 and 52% of TAR level 2 and a mean glucose of 257 mg/dL **(b)** Follow-up, Visit 4 CGM AGP. The report from July-August 2023 shows significant improvements, with Time in Range increasing to 63%, mean glucose reduced to 164 mg/dL, and stable glucose variability (33.5%). **(c)** Glycemia Risk Index (GRI) progression over four follow-up visits. The patient’s profile moved from Zone E (visit 1, high hyperglycemia risk) to Zone B (visit 4, lower risk), indicating a marked improvement in glycemic control

At the first follow up visit, CGM data showed poor glycemic control (Fig. 1a), and analysis of the food diary revealed inadequate adherence to the PKU diet, due to the T1D diagnosis, as the patient preferred conventional whole grain products and animal protein sources to manage post-prandial hyperglycemia; this resulted in an increased dietary phenylalanine intake and consequently elevated phenylalanine levels (Phe 842 µmol/L); moreover, a brain MRI indicated signs of metabolic accumulation distress, characterized by white matter hyperintensities typically associated with chronic elevation of brain phenylalanine levels.

To address these challenges, the patient was transitioned from fixed insulin doses to a dynamic regimen. He was educated on “Carbohydrate Counting” specifically adjusted for PKU-specific medical products. A new PKU-adapted diet was prescribed, featuring special low-protein foods (SLPF) low in refined sugars and maltodextrins and with phe-free aminoacid supplements without added sugars. The medical nutrition therapy (MNT) was normocaloric, targeting 500 mg/day of Phe and a protein intake of 1 g/kg/day. The diet is essentially plant-based (excluding all animal protein sources) and includes low-protein foods (low-protein/protein-free pasta and bread). Therefore, to achieve a normal protein intake of 1 g/kg/day, phe-free amino acid supplements are necessary. The MNT has been set up with 80% of total protein from supplements, “special” foods and foods naturally characterized by a low protein content (fruit and vegetables with Phe ≤ 75 mg/100 g) and the remaining 20% of total protein was provided in the form of large neutral amino acid (LNAA) that reduces Phe accumulation in the brain by limiting its passage through the blood-brain barrier, competing for the same transporter.

Before reintroducing PKU medical nutrition therapy, to further stabilize post-prandial glucose,the patient was instructed to maintain a specific pre-bolus interval of 10 min when using ultra-rapid insulin and to include fiber-rich vegetables at each main meal to lower the overall glycemic index. The shift from fixed dosing to a personalized insulin-to-carbohydrate ratio (ICR), determined through a two-week glycemic-dietary diary, allowed for a more precise management of the glycemic load. This multidisciplinary approach resulted in a significant improvement in HbA1c (7.8% vs. 11.5% at baseline), Phe levels (705 µmol/L vs. Phe 842 µmol/L at baseline), and overall glycemic control over the first four months. At visit 4, CGM AGP confirmed this positive trend (Fig. 1b), with an improved Time in Range, reduced Time Above Range, and a lower mean glucose, without increasing hypoglycemia. At the same time, insulin requirement decreased from 0.55 IU/kg per day to 0.3 IU/kg per day.

In addition, the Glycemia Risk Index (GRI) improved from Zone E (high risk) to Zone B (low-intermediate risk), as shown in Fig. 1c. Of note, the improvement in GRI was concomitant with stably reduced Phe levels.

## Discussion and Conclusion

The coexistence of PKU and T1D represents clinical and nutritional challenges. This case report illustrates the inherent conflict between the dietary management of a disorder requiring protein restriction (PKU) and another disorder in which management of carbohydrate intake is key (T1D). Our experience aligns with the limited existing literature, which describes similar difficulties in maintaining metabolic control for both conditions simultaneously. A key apparent challenge was the patient’s behavioral response to the new T1D diagnosis, where the acute need to manage hyperglycemia led to a de-prioritization of the chronic, less symptomatic PKU dietary restrictions. A cornerstone of our intervention involved personalized therapeutic education, which focused on teaching the patient to incorporate carbohydrate counting into his PKU diet, and the introduction of modern medical foods, such as low-sugar SLPF and sugar-free formulas.

Living with chronic conditions has a significant emotional impact. It is well-known that individuals affected by chronic diseases like PKU and T1D are more susceptible to developing eating disorders, likely due to the lifelong adherence required for stringent dietary recommendations. Achieving therapeutic goals is indeed contingent on the patient’s adherence to the multiple and restrictive nutritional therapeutic recommendations that characterize both conditions (e.g., taking a protein substitute at least three times a day and insulin with every meal; sourcing special nutritional products and therapeutic aids; periodic clinical-laboratory monitoring). Therefore, it is fundamental to ensure these patients constant psychological support that should be offered through all phases of their lives, and particularly during the delicate phase of transition from pediatric care centers to adult care centers.

Recognizing the significant psychological impact of managing these two conditions, the patient was advised to pursue specialized psychological counseling. This recommendation was complemented by the multidisciplinary team’s strategy of providing intensive follow-up and constant clinical support, as an essential path for building a strong therapeutic alliance and empowering the patient in his daily self-care.

A single-case study has inherent limitations, as its findings are not broadly generalizable. However, it may provide valuable insights into managing this rare dual diagnosis. Future research is advisable, perhaps through the establishment of multicenter registries and the systematic collection of data on the prevalence, clinical challenges, and long-term outcomes of this unique patient population. Further investigation into the development of PKU-specific medical foods with a low glycemic index could also offer significant therapeutic benefits.

**Fig. 2:**
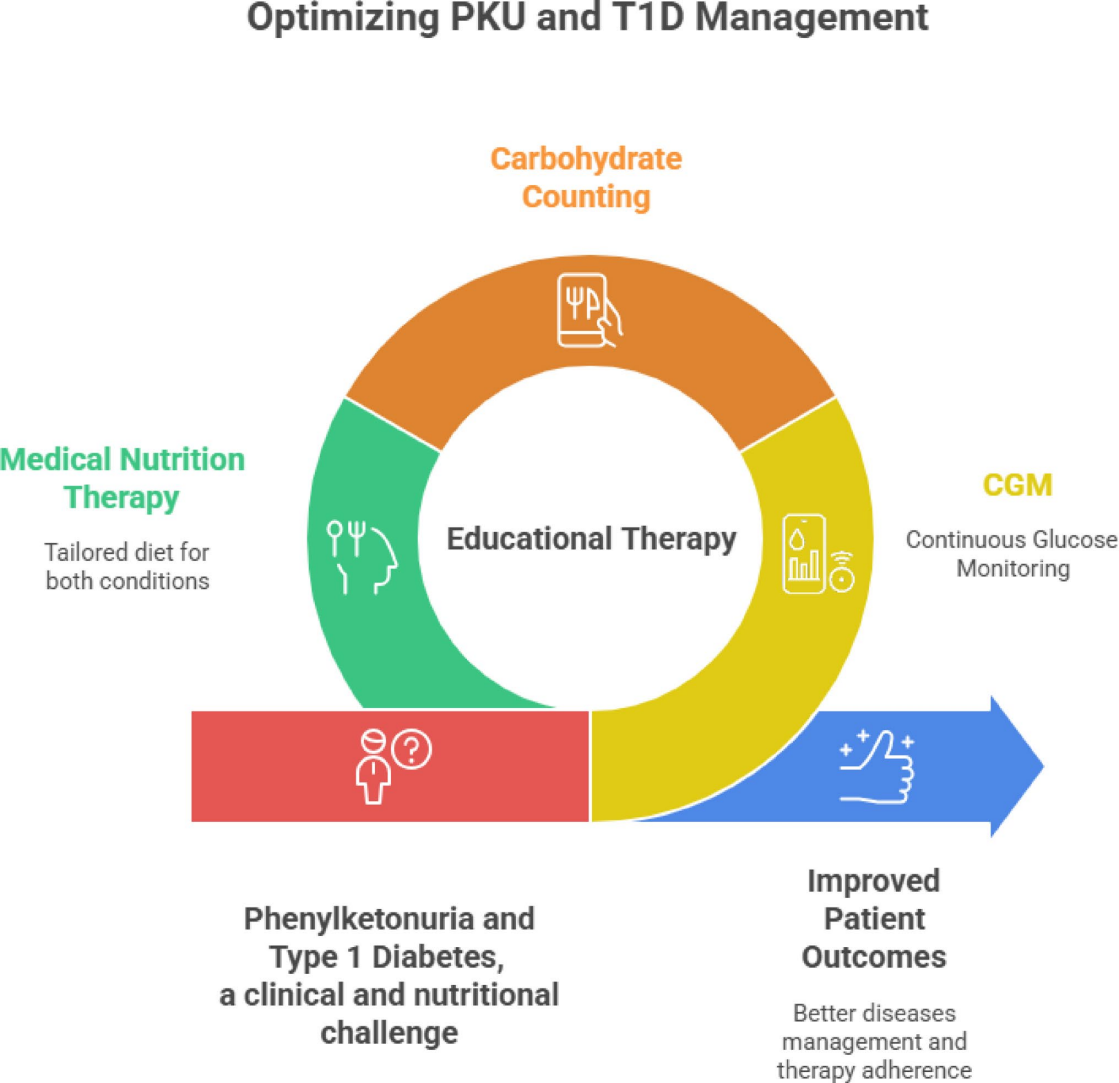
Integrated educational therapy, continuous glucose monitoring, carbohydrate counting and tailored nutrition together optimize the management of PKU and Type 1 Diabetes, leading to improved patient outcomes and better adherence to therapy

The coexistence of PKU and T1D in a young adult is a complex clinical challenge, requiring an integrated and personalized therapeutic approach (Fig. 2). This case highlights the importance of multidisciplinary management and CGM-driven insulin therapy and education to optimize metabolic control of both conditions and improve the patient’s quality of life.
